# Euro VI-d Compliant Diesel Engine’s Sub-23 nm Particle Emission

**DOI:** 10.3390/s23020590

**Published:** 2023-01-04

**Authors:** Norbert Biró, Péter Kiss

**Affiliations:** 1 IBIDEN Hungary Kft. Technical Center, Exhaust System Evaluation, 2336 Dunavarsány, Hungary; 2 Department of Vehicle Technology, Institute of Technology, Hungarian University of Agriculture and Life Sciences, 2100 Gödöllő, Hungary

**Keywords:** Euro 7 regulation, condensation particle counter sensor, sub-23 nm particle emission, commercial transportation emission

## Abstract

Passenger and commercial transportation significantly contribute to hazardous air pollution. Exhaust gas after-treatment technology advances closely to the emission regulations throughout the world. The upcoming legislation will be EURO VII in European Union, which requirements are not set yet, but the Solid Particle Number (SPN) diameter range is expected to be more severe compared to EURO VI. This paper will revisit the measurement principle differences between over 10 nm and over 23 nm diameter particles in theory and practical engine bench measurement. Two different types of particle counters have performed the soot particle counting measurement; therefore, the applied sensors are different in terms of applied counting principles. The measurement principles of both devices will be introduced, and the experiment’s result will reflect on the sensor differences. From this, a conclusion can be derived in order to determine the severity of the upcoming EURO VII legislation in terms of SPN, and the experiment will also reflect on the measurement sensor differences. The overall results suggested that extending the lower range of the measurement increases the tailpipe particle emission by 20%, although the DPF filtration efficiency is still over 99%.

## 1. Introduction

The reduction of harmful emissions of a great interest in both scientific and public circles. The average of the Euro 27 countries attributes 14% of GHG emissions to transport [1]; therefore, further emission reduction possibilities have to be pursued. The total electricity produced in the world in 2016 was about 25 PWh, two-thirds of which came from fossil energy carriers. Numerous well-to-wheel analysis determines lower overall emission for diesel-powered ICE drive trains than the BEVs [2]. Bearing these statements in mind, internal combustion engines still play an important role in the commercial vehicles sector, economy-wise and from an environmental standpoint. It should be noted that breakthroughs in ICE development are ongoing [3]. Therefore investing in further studies are necessary to have a better understanding of the emission processes. Exhaust gas after-treatment technology advances closely to the emission regulations throughout the world. The most stringent regulation is the European legislation to date; the rest of the world is using it as a baseline. The upcoming legislation will be the Euro VII, which requirements are not set yet, but the particle number measurement’s lower diameter range is expected to be more severe compared to the Euro VI [4]. The size of aerosol (exhaust gas composition) particles ranges from the smallest stable molecular agglomerations [5], around 0.7 nm in diameter, to solid abrasively generated particles, around 10 µm in diameter.

**Figure 1:**
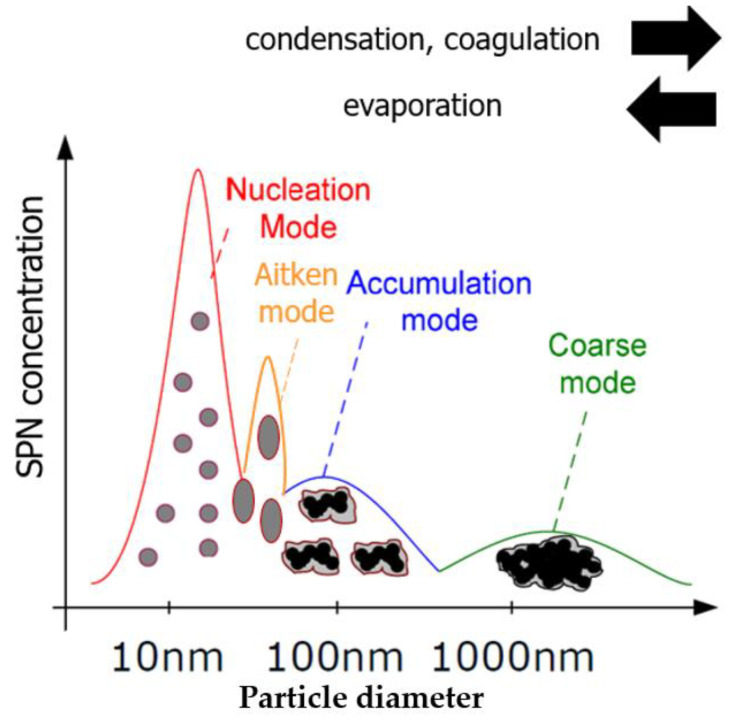
Particle size distribution over modes; SPN = Solid Particle Number.

The particle diameter distribution originated from different formation methods, often called modes. The categorization of the modes and particles sizes is continuously changing over time. It is mainly because, on the microscopic level, the aerosol is rapidly and volatilely flowing. Therefore, on the macroscopic level the thermodynamic state is ever-changing and not in equilibrium. In other words, evaporation, coagulation, and condensation are responsible for most of these mode and diameter transformations. In Figure 1, an overview can be seen, showing the different modes and formations. The distinguishable modes are Nucleation mode, Aitken mode, accumulation mode, and coarse mode in order of increasing size [6]. The nucleation mode is the smallest diameter class, formed by gases that convert to particles; this mode is transitional, usually rapidly growing into larger particles due to condensation and dissipation. The Aitken mode particle size stretches between 10–50 nm. The formation can be either because of enlarged nucleation particles or by non-stochiometrical ideal combustion (soot particles). The accumulation mode particle size is between 50–100 nm and the transition time in this mode is higher than the before mentioned. The formation can be because of coagulation and condensation of different mode particles and by combusting non-combustible substances (e.g., engine oil). Coarse mode particles are mainly formed by coagulation of accumulation particles; the diameter extends from 100 nm to 10 µm. Due to their size, the coarse mode particles are greatly affected by friction and abrasion.

The mode size distribution is not uniform, and the variation is subject to the combustion strategy, fuel, engine oil, and intake air humidity. The distribution of modes is greatly affected by the fuel type. Currently, 7% of diesel fuel contains biodiesel, but in the future it may increase, since this fuel type is considered to be renewable. Abdulfatah Abdu Yusuf and his team recently examined the emission and the particle size distribution penetration shifts by different biodiesel fuels and additives [7].

The Solid Particle Number (SPN) was originally introduced in the Euro 5 for the Light Duty (LD) compression ignition vehicles under the NEDC cycle. The new legislation requirement could not be met by optimizing the in-engine processes; adding a high filtration efficiency porous particle filter was necessary to satisfy the legislation requirement. Starting from the Euro VI (Euro 6 for LD), the legislation prescribes 6∙10^11^ #/km for the LD and 6∙10^11^ #/kWh for the Heavy Duty (HD) compression ignition vehicles [8]. The same standard was introduced for gasoline direct injection at Euro VI-d-Temp in September 2017.

The most widespread method of particle number determination in aerosol is condensation particle counting. In this method, the solid particles of the aerosol are enlarged by dissipating liquid butanol first. Then the particles pass by laser light and light-sensing optics in a detection chamber [9]. The passing of the enlarged particles causing discontinuity in the laser detection of the optics, allowing the particle count to be determined.

In solid particle measurement, sample gas preparation is an important task. For the measurement of solid particles over 23 nm, an evaporation tube (ET) is necessary, while for those over 10 nm, a catalytic stripper (CS) is needed [10]. Both ET and CS heat the sampled gas over 350 °C, so the downstream liquids evaporate, and only the solid particles remain. Many of these principles originated from the particle measurement program [11]. The main reason for the sudden interest from the European Commission is probably due to the severe health effects caused by sub-23 nm particles [12]. Although nucleation mode particles rapidly evolve to different modes, in today’s cities, during that short lifetime, they can reach the human body. Numerous research examined the untreated raw exhaust gases’ PN10 concentration [13], while the real interest lies in whether the tail-pipe emission of a Euro VI-compliant vehicle after treatment system (ATS) can satisfy the possible Euro VII requirements or not. Naturally, papers have been published where tail-pipe emission has been measured involving sub-23 nm particles. Giechaskiel and his team [14] conducted an extensive experimental series where gasoline and Euro 6 b diesel passenger car’s sub and over 23 nm solid particles distribution were compared. The results showed around a 20% deviation between the two size class. This paper will only focus on the Euro VI-d heavy-duty diesel engines since, as stated earlier, unlike the passenger car segment, long-distance transportation will rely on diesel technology and remains important in the future. Testing the latest HD engine (Euro VI-d ISC-FCM introduced on 1 January 2021) technology with the cutting edge HP-DPF (high porosity diesel particle filter) will provide an approximation about the development pressure on HD ATS and DPF manufacturers for the upcoming, possibly the last ICE (internal combustion engine) regulation in the EU. In order to ensure that the experiment returns reliable data, two measurement layouts were pre-tested, and the most suitable was selected. The engine bench experiment also includes raw and tail-pipe measurements, to compare the distribution of PN10 and PN23 and determine DPF filtration efficiency.

## 2. Materials and Methods

The tests were performed in the Exhaust System Evaluation department of Ibiden Hungary’s Technical Center. An AVL HD 500 kW engine dynamometer test cell (AVL List GmbH, Graz, Austria) was used with fully equipped engine fluid conditioners. The sample gas was diluted with fine-filtered compressed air on every test layout. Two SPN measuring layouts were considered.

### 2.1. Firstly Considered Measurement Layout

**Figure 2:**
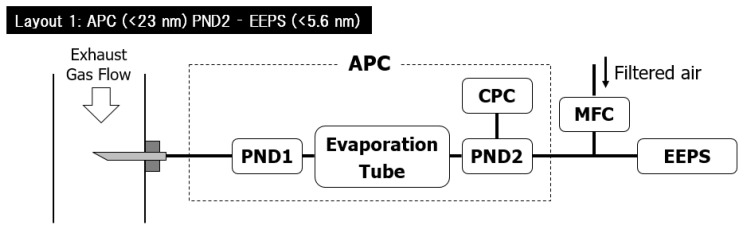
Considered measurement layout consisting of an APC and an EEPS. APC = AVL Particle Counter; CPC = Condensation Particle Counter; PND = Particle Number Diluter; MFC = Mass Flow Controller; EEPS = Engine Exhaust Particle Sizer.

Firstly the AVL condensation particle counter (APC 489 plus) (AVL List GmbH, Graz, Austria) for over 23 nm and the Engine Exhaust Particle Sizer (EEPS 3090) (TSI Incorporated, Minneapolis, MN, USA) for over 10 nm particle measurement was considered. In this layout, the dilution and the volatile particle removal (VPR) have been carried out by the APC, and the excess sample gas has been fed to the EEPS, as seen in Figure 2. Dilutor 1 and 2 are abbreviated to PND1 and PND2, while the MFC stands for Mass Flow Controller (TSI Incorporated, Minneapolis, MN, USA).

The EEPS spectrometer (TSI Incorporated, Minneapolis, MN, USA) uses an array of sensitive electrometers, while the APC visually detects particles that have been enlarged by condensed butanol. Due to the inherently different measuring principles, initial tests were necessary to confirm whether the two devices were able to measure in combination. The overlapping conditions where both devices’ counting efficiency is satisfactory are quite small and cannot be considered for extensive experimental work.

**Figure 3:**
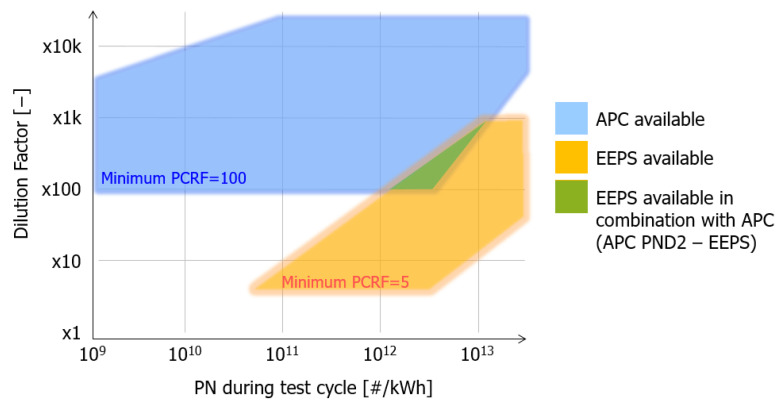
Overlapping dilution factor areas of APC and EEPS. APC = AVL Particle Counter; CPC = Condensation Particle Counter; PND = Particle Number Diluter; EEPS = Engine Exhaust Particle Sizer; PCRF = Particle number concentration reduction factor.

In Figure 3, the applicable dilution factor has been shown in relation to the PN calculated final result of individual tests. The overlapping green area is where both devices can work sufficiently in combination.

### 2.2. Secondly Considered Measurement Layout

The first layout’s initial idea was to use the same thermodilution and VPR-treated sample gas for both measuring devices to increase the comparability. Although because of the different working principles, the first layout turned out as non-ideal, the base concept was right. To overcome the issues of the first layout, in the second layout, both devices have the very same working principle. The dilution and volatile particle removal were done by an APC (APC Plus Advanced 10 nm) (AVL List GmbH, Graz, Austria), but unlike in the previous layout, here, the APC is enabled for 10 nm measurements and includes a catalytic stripper (CS) for increased VPR effect. The secondary here is a standalone AVL condensation particle counter (CPC 488), which does not feature any sample gas preparation unit. For the 23 nm and over measurement, the ET has been used for VPR, which could be a conflict since the CPC receives a gas treated by the CS.

**Figure 4:**
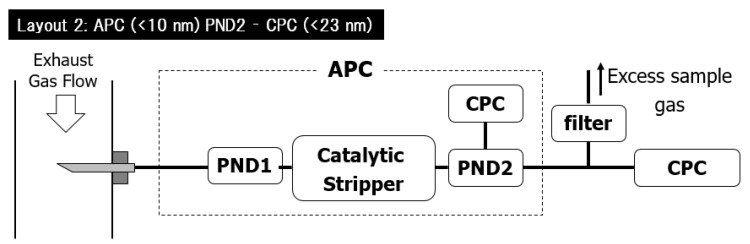
Considered measurement layout consisting an APC and a CPC. APC = AVL Particle Counter; CPC = Condensation Particle Counter; PND = Particle Number Diluter.

Christoph Kandlhofer of AVL [15] has made a comparison experiment, which concluded that the <23 nm measurement sample gas could be treated either in ET or CS; the deviation is within −6% to +1%. As shown in Figure 4, the excess sample gas is routed to the ambient air of the test cell after filtering. The outgoing gas volume flow of the APC is 9 l/min, while the CPC requirement is only 1 l/min; therefore, the excess air outlet is mandatory to protect the devices from overpressure. This configuration of measurement instruments gave reproducible and reliable results during the initial measurements; therefore, it has been chosen for further experiments.

### 2.3. Instrumentation Calibration

The AVL APC489 (AVL List GmbH, Graz, Austria)and the CPC 488 (AVL List GmbH, Graz, Austria)are subject to annual calibrations [16] performed before the experiments. All the calibrations were carried out by the manufacturer. The particle number calibration was performed with a condensation particle counter as a reference, which complies with the UN/ECE GTR 15. The measurement uncertainty check has been performed according to the “Guide to the expression of uncertainty in measurement” [17]. The overall measurement certainty is over 95%.

### 2.4. Test Protocol

All the tests were carried out in an engine dynamometer laboratory environment. The AVL emission automation system adjusts the experiments to the official WHTC test cycle requirements. The unit under test was a Euro VI-d compliant six-cylinder, heavy-duty diesel engine. The World Harmonized Stationary Cycle (WHSC) was used throughout all of the tests; this is a stationary engine dynamometer schedule defined by the global technical regulation (GTR). The regulation is based on the worldwide pattern of real heavy commercial vehicle use. The modes of WHSC have been de-normalized [18] to this specific test engine by the emission automation software called AVL PUMA Open^TM^. The overview of the test protocol can be seen below [19]:

- WHSC mode 9 precondition—125 s
- Soaking—300 s
- WHSC—1895 s

The advantage of the stationary engine conditions over transient is the better comparison since all the output variables are nearly steady, while the transient cycle outputs are harder to compare, although they are closer to real-life usage.

**Figure 5:**
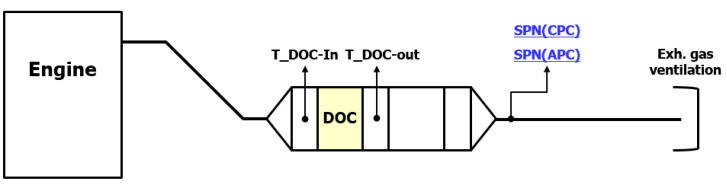
Exhaust gas aftertreatment system layout consisting a DOC. DOC = Diesel Oxidation Catalyst; SPN = Solid Particle Number; APC = AVL Particle Counter; CPC = Condensation Particle Counter.

**Figure 6:**
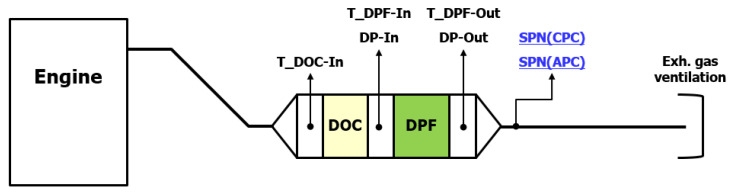
Exhaust gas aftertreatment system layout consisting a DOC and a DPF. DOC = Diesel Oxidation Catalyst; DPF = Diesel Particle Filter; SPN = Solid Particle Number; APC = AVL Particle Counter; CPC = Condensation Particle Counter.

The tests were divided into with and without DPF measurements. The most favorable layout would be the simultaneous particle number measurement upstream and downstream of the DPF [20]. Unfortunately, it is not feasible with the available number of measurement devices. Therefore the after-treatment system (ATS) layout 1 (Figure 5) corresponds to the upstream measurement while the 2nd ATS layout (Figure 6) to the downstream. This layout has been tested and proved in a previous experiment, where a self-designed adjustable automatic Adblue injection system (Ibiden Hungary, Dunavarsány, Hungary) tested [21].

In Figure 6, the installed DPF is enabled for the latest Euro VI-d applications, so the results are expected to be used for future considerations.

### 2.5. Calculations

All calculations were based on the UN/ECE guidelines; therefore, the calculated results are comparable to the Euro emission standards [22] and PN requirements. The exhaust volume flow is the sum of the intake air and the fuel volume flow. Multiplying it with the dilution-corrected particle number count equals the total particle number count of the cycle. For all tests that adopted the exhaust layout of Figure 5, a dilution factor of 20,000 was applied. It was necessary to prevent clogging in the emission measurement devices since no particle reduction solution was implemented in this layout. On the other hand, for the Figure 6 layout, considerably lower dilution was sufficient; the applied factor was 2000. The final solid particle number can be calculated by dividing the total PN by the work done by the engine in the WHSC cycle.

(1) $$SPN=\frac{EVF\cdot PN}{P}\;\left[\#/kWh\right]$$

EVF = Exhaust volume flow over the WHSC cycle [cm^3^]; PN = average PN count over the WHSC cycle [#/cm^3^]; P = work done over the WHSC cycle [kWh]; SPN = Solid particle number [#/kWh].

(2) $${\mathrm{FE}}_{10}=1-\left(\frac{{\mathrm{PN}}_{\mathrm{dpf}10}}{{\mathrm{PN}}_{\mathrm{raw}10}}\right)\cdot 100\;{\left[\%\right]}_{\;}$$

FE_10_ = DPF 10 nm particle filtration efficiency; PN_dpf10_ = Average 10 nm solid particle result of the 3 tests with DPF; PN_raw10_ = Average 10 nm solid particle result of the 3 tests without DPF.

(3) $${\mathrm{FE}}_{23}\;=1-\left(\frac{{\mathrm{PN}}_{\mathrm{dpf}23}}{{\mathrm{PN}}_{\mathrm{raw}23}}\right)\cdot 100\;\left[\%\right]$$

FE_23_ = DPF 23 nm particle filtration efficiency; PN_dpf23_ = Average 23 nm solid particle result of the 3 tests with DPF; PN_raw23_ = Average 23 nm solid particle result of the 3 tests without DPF.

## 3. Results

### 3.1. Raw Emission

Firstly, three tests were conducted without DPF (Figure 5). The six individual SPN (3 pcs PN10; 3 pcs PN23) test results have been checked at each WHSC mode, and the calculated final results have been compared.

**Figure 7:**
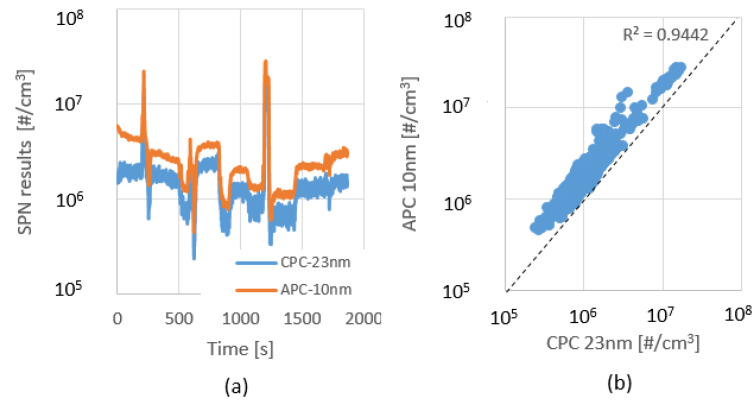

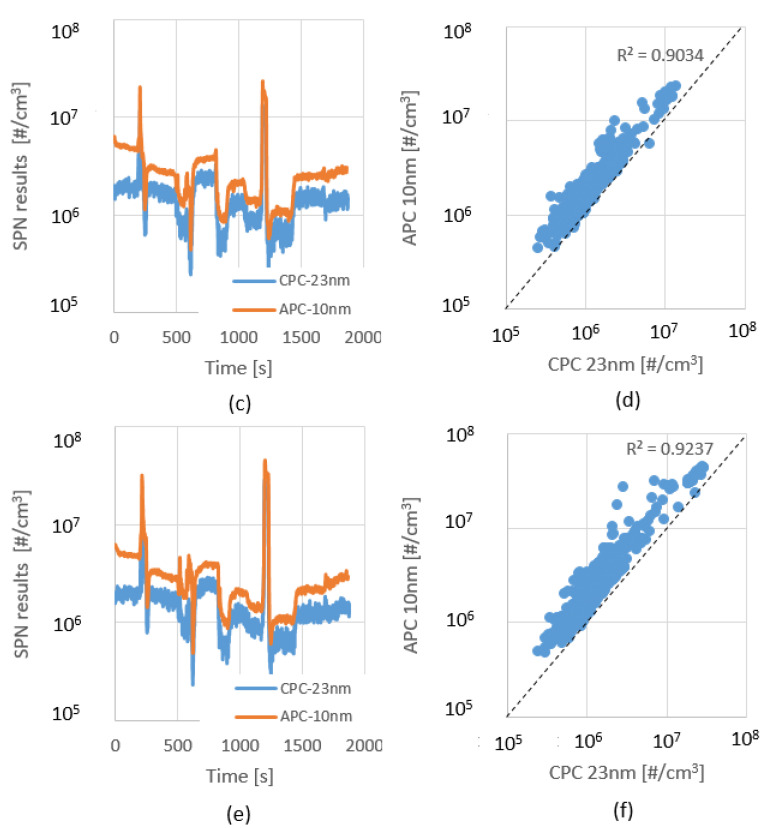
Transient particle concentration over 1st (**a**); 2nd (**c**); 3rd (**e**) WHSC cycle; Proportion of variance between <10 nm and <23 nm results over 1st (**b**); 2nd (**d**); 3rd (**f**) WHSC cycle. APC = AVL Particle Counter; CPC = Condensation Particle Counter; SPN = Solid Particle Number.

**Figure 8:**
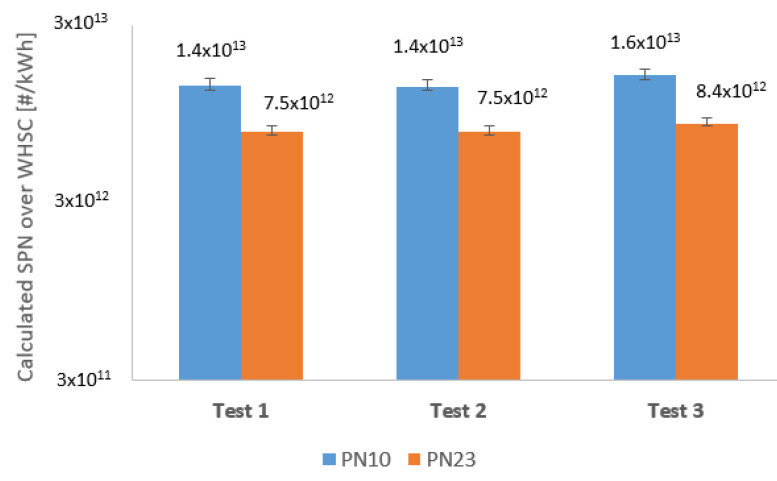
Calculated solid particle number results of each individual test, without DPF.

In order to draw a reliable conclusion, every test was repeated three times. Every test has been examined by the transient particle concentration over gas volume (Figure 7), R-Squared in a regression model that determines the proportion of variance (Figure 8), and by UN/ECE approved calculations which have been discussed in Section 2.5.

It can be seen in Figure 7 that the CPC follows APC’s curve, and the offset is normal deviation due to the different measurement ranges. However, the CPC has a certain noise in its signal, which is due to the different lower detection limits of the two devices.

Overall, the deviation from <10 nm to <23 nm measurements are uniform. The R squared value is close to 1, although by the right side graph, it seems the points are far from it. That is because the distribution of the points is different, but the majority of the results are close to the dashed line.

On the one hand, this proves the reliable repeatability of the test series, while on the other, it shows a considerably higher particle emission if we include the sub-23 nm particles in the measurement.

The <10 nm and the <23 nm relation will be the baseline to compare with the high-porosity DPF tests.

### 3.2. Tail Pipe Emission

**Figure 9:**
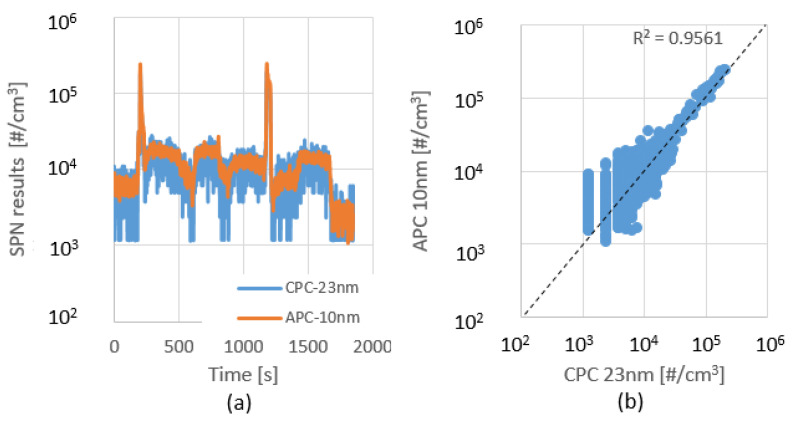

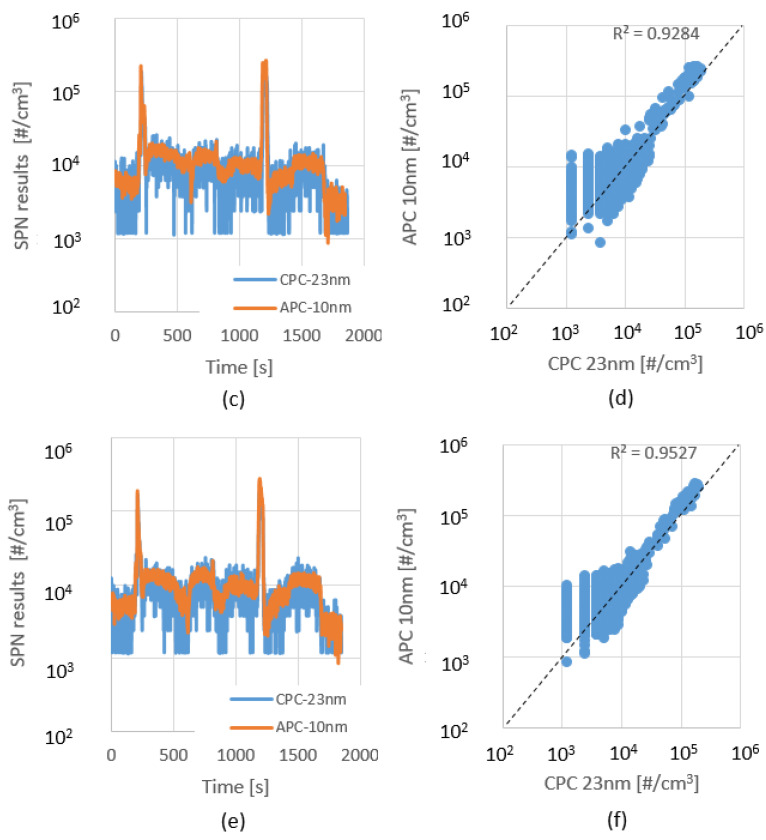
Transient particle concentration over 1st (**a**); 2nd (**c**); 3rd (**e**) WHSC cycle; Proportion of variance between <10 nm and <23 nm results over 1st (**b**); 2nd (**d**); 3rd (**f**) WHSC cycle. APC = AVL Particle Counter; CPC = Condensation Particle Counter; SPN = Solid Particle Number.

In order to determine the tail-pipe emission, during the following tests, a high porosity Euro VI-d compliant DPF was in use. The measurement layout, which has been explained in detail in Section 2.4 and shown in Figure 6, was applied. The test protocol was the same as in the raw emission test. A similar fluctuating behavior was observed (Figure 9) as seen in the previous test series (Figure 7), although the emission levels were much lower in this case.

If the peak values are reviewed, there are two orders of difference, 10^5^ [#/cm^3^] vs. 10^7^ [#/cm^3^]. In addition, the APC-CPC particle count distribution is more balanced, around the R^2^ = 0.95. As the particle concentration lowers, the CPC signal’s affinity for fluctuations increases.

**Figure 10:**
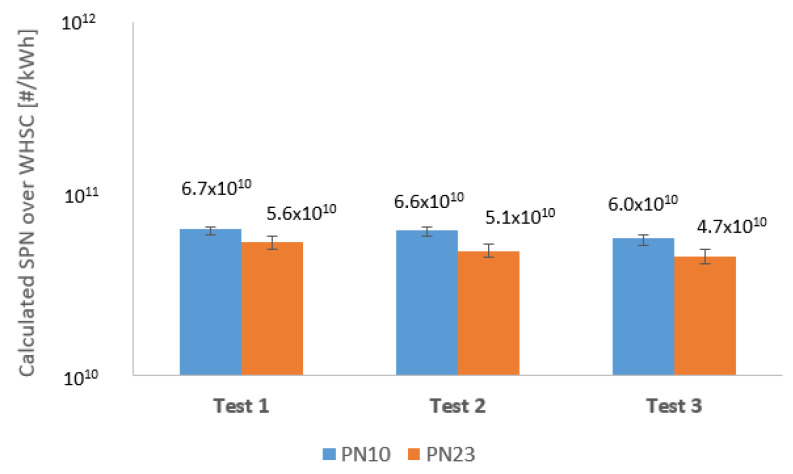
Calculated solid particle number results of each individual test, with DPF.

The calculated particle count is much closer compared to the size classes, and each of them is much lower than legislation limit as it is seen in Figure 10. Knowing the upstream and downstream calculated PN of the DPF, the filtration efficiency is easy to determine by applying Equations (2) and (3). The with and without DPF results in both size classes are averages of the 3–3 test series.

$${\mathrm{FE}}_{10}\;=1-\left(\frac{1.4\;\cdot \;10^{13}}{6.4\;\cdot \;10^{10}}\right)\cdot 100=99.5\;\left[\%\right]$$

$${\mathrm{FE}}_{23}\;=1-\left(\frac{7.8\;\cdot \;10^{12}}{5.1\;\cdot \;10^{10}}\right)\cdot 100=99.3\;\left[\%\right]$$

Seeing the result, it is apparent that the DPF filtration efficiency is quite high, only around 0.5% of the particles are emitted to the ambient air.

## 4. Discussion

### 4.1. Euro VI-d Compliant Engine Raw Exhaust Gas Particle Size Distribution

The performance of the 10 and 23 nm measurements without DPF layout is summarized in Table 1. The average range of difference of 10 and 23 nm (Diff.) is between 45%+1. That means the raw particle emission is greatly influenced by extending the lower measurement range to 10 nm. The most likely explanation is that modern common rail engines, such as the unit used throughout this test series, apply high fuel injection pressures. This high pressure (over 2500 bar) causes the formation of the sub-23 nm particles in higher numbers.

### 4.2. Euro VI-d Compliant DPF Exhaust Gas Particle Size Distribution

The performance of the 10 and 23 nm measurements with DPF layout is summarized in Table 2. The average range of the difference of 10 and 23 nm (Diff.) is between 20% +3. This is a very similar result as the Sustainable Transport Unit of the European Commission discovered in 2017 [14]. The uniformity of the results, in this case, is not as good as the raw measurements. However, the proportion shifted between PN 10 and 23.

The usage of the latest DPF not only reduces the sum number of particle emissions but also decreases the ratio of more harmful sub-23 nm particles. The CPC reached its lower counting range with the high filtration DPF; therefore, 0 can be seen at the minimum value of PN 23.

### 4.3. Calculated DPF 23 nm PN Results Comparing to EURO VI

The PN emission of <23 nm particles was found to be lower than the Euro 6 regulation limit.

**Figure 11:**
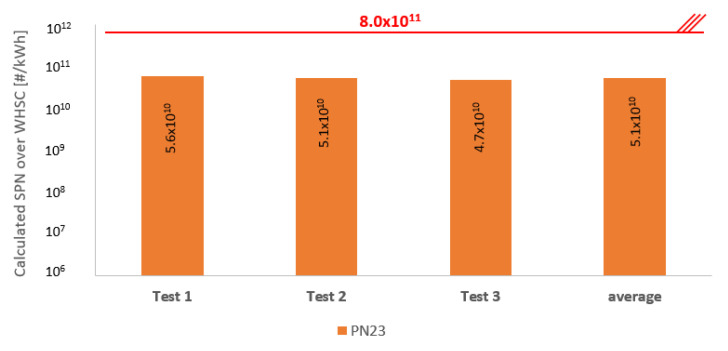
Calculated solid particle number results of each individual test, with DPF compared to Euro VI requirement.

This finding was as expected since the engine and the DPF comply with the Euro VI-d legislation inherently. However, the measured results are more than one order lower than the legislation limit, as it is seen at Figure 11.

### 4.4. Calculated DPF 10 nm PN Results Comparing to EURO VII Proposal

During the editing of this article, a proposal [23], which is likely to be effective is introduced by the European commission. In this proposal, as the industry anticipated, the sub-23 nm particles are involved. Therefore there is a chance to compare the PN 10 results of this research to the proposed limit.

**Figure 12:**
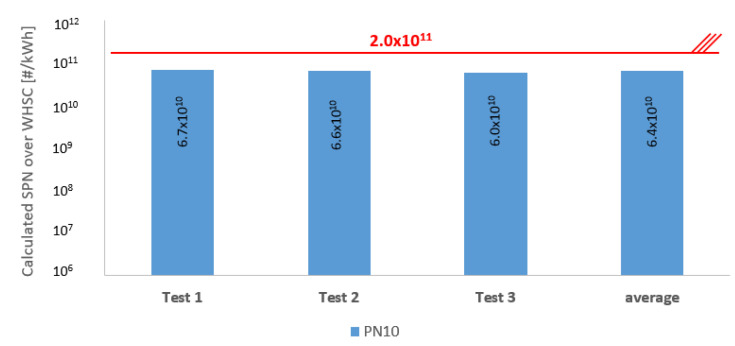
Calculated solid particle number results of each individual test, with DPF compared to Euro VII proposal; * 2.0 × 10^11^ = Euro VII proposal PN10 limit.

All the measured results are under the legislation, but the gap has become much smaller as it is seen at Figure 12.

The most important aspect of particle measurement is aerosol preparation, such as dilution, filtering out coarse particles, condensing and removing excess vapor and removing volatile particles. This study revealed not many differences between the ranges APC and CPC, despite distinct measuring ranges. The results underline that the reproducibility of both devices is excellent. To ensure that proper aerosol preparation has to be taken care of, even in raw measurement mode.

## 5. Conclusions

Knowing a possible PN limitation for future Euro VII legislation allowed us to see how current particle reduction technology performs in light of the new requirement. The average of the tail-pipe experiments was 6.4∙10^11^ #/kWh, which is 68% lower than the recently proposed SPN requirement of Euro VII. The overall filtration efficiency of the HP-DPF is still satisfactory, over 99%, even when the PN10 nm is considered. Comparing the raw and tail-pipe SPN, there is a considerable gap. With the latest high injection pressure engine technology, overall raw SPN output decreased, but the distribution shifted towards the sub-23 nm particles, representing an even greater health concern than the larger particles.

The data suggesting the current after-treatment systems can satisfy the new requirement. Although further, more versatile experiments are necessary, especially cold and hot start transient cycles such as WHTC, since those are historically creating higher emissions.

## Figures and Tables

**Table 1:** Comparing particle count of the without DPF tests.

		Test 1	Test 2	Test 3	
PN 10	Max	28,357,030.0	23,164,458.0	45,137,712.0	[#/cm^3^]
	Min	453,430.0	448,496.0	488,879.0	[#/cm^3^]
	Ave	2,913,247.7	2,903,720.8	3,409,744.4	[#/cm^3^]
PN 23	Max	16,866,000.0	13,256,000.0	28,796,000.0	[#/cm^3^]
	Min	234,000.0	248,000.0	234,000.0	[#/cm^3^]
	Ave	1,598,389.4	1,607,762.5	1,832,046.4	[#/cm^3^]
Diff.	Max	40.5	42.8	36.2	[%]
	Min	48.4	44.7	52.1	[%]
	Ave	45.1	44.6	46.3	[%]

**Table 2:** Comparing particle count of the with DPF tests.

		Test 1	Test 2	Test 3	
PN 10	Max	241,959.0	266,646.0	282,673.0	[#/cm^3^]
	Min	1108.0	854.0	855.0	[#/cm^3^]
	Ave	14,661.2	14,437.2	13,109.6	[#/cm^3^]
PN 23	Max	197,800.0	184,200.0	194,000.0	[#/cm^3^]
	Min	0.0	0.0	0.0	[#/cm^3^]
	Ave	12,350.2	11,030.8	10,387.9	[#/cm^3^]
Diff.	Max	18.3	30.9	31.4	[%]
	Min	-	-	-	[%]
	Ave	15.8	23.6	20.8	[%]

## Data Availability

The data presented in this study are available on request from the corresponding author. The data are not publicly available due to the research data can aid competitors of the company where the experiments were carried out.

## Abbreviations

APC	AVL particle counter
ATS	After treatment system
DPF	Diesel particle filter
SPN	Solid Particle number
LD	Light Duty
HD	Heavy Duty
ICE	Internal combustion engine
WHSC	World harmonized stationary cycle
NEDC	New European driving cycle
CPC	Condensation particle counters
CS	Catalytic stripper
DPF	Diesel particulate filter
HP-DPF	High porosity diesel particulate filter
EEPS	Engine exhaust particle sizer
ET	Evaporation tube
PCRF	Particle number concentration reduction factor
PMP	Particle measurement program
PN	Particle number
PND	Particle number diluter
TP	Tailpipe
UNECE	United Nations Economic Commission for Europe
GHG	Greenhouse gas
